# Complete mitochondrial genome of *Sellanucheza jaegeri* Golovatch, 2013 by next generation sequencing (Polydesmida: Paradoxosomatidae) and phylogenetic analysis in Diplopoda

**DOI:** 10.1080/23802359.2018.1473729

**Published:** 2018-05-18

**Authors:** Chao Yang, Xue-Juan Li, Hao Yuan, Jian Shen, Mei-Xia Yang

**Affiliations:** a Shaanxi Institute of Zoology, Xi’an, China;; b School of Life Sciences, Shaanxi Normal University, Xi’an, China

**Keywords:** *Sellanucheza jaegeri*, mitogenome, Diplopoda, phylogeny

## Abstract

The mitogenome of *Sellanucheza jaegeri* was 15,623 bp long, revealed the same gene order to that of typical Polydesmida. Both the *tRNA^Ser^(AGN)* and *tRNA^Ser^(UCN)* lacked the DHU arms. No tandem repeat was found in two control regions. Phylogenetic analysis indicated that Sphaerotheriida was so antiquity that divided out earlier than others. We supported that Polydesmida had a relatively systematic affinity between Julida and Playtdesmida, and suggested that the interordinal phylogenetic relationships within Diplopoda should be further investigated.

Diplopoda (millipedes) is the third most diverse class of Arthropoda, have diversified for over 400 million years, with ∼12,000 species described and an estimated diversity of 80,000 living species (Zhang 2011; Brewer et al. 2012; Enghoff et al. 2015). They occupy an important niche in the ecosystem because of their function in the breakdown of organic matter. The rearrangement of genes in mitogenomes had remarkably occurred in Diplopoda. However, relationships among the Myriapoda remain unresolved in Robertson et al. (2015), and owning to the lack of species sequences, the interordinal phylogenetic relationships within Diplopoda are one of the most debated issues.

Sample (voucher no. MP06) of *Sellanucheza jaegeri* was collected from Feng County (34°12'45''N, 106°54'09''E), Shaanxi, China. Genomic DNA was prepared in paired-end libraries, tagged and subjected to the Illumina Xten platform (Illumina, San Diego, CA) with 150 bp paired-end strategy, and yield 16,147,682 paired-end raw reads. Mapping against the complete mitogenome of *Appalachioria falcifera* (GenBank: JX437063), high-quality reads were assembled using MITObim version 1.9 (University of Oslo, Norway) (Hahn et al. 2013). A total of 201,061 individual mitochondrial reads gave an average coverage of 1879.4X. Comparing with the *Asiomorpha coarctata* (GenBank: KU721885), annotations were generated in MITOchondrial genome annotation Server (MITOS) (Bernt et al. 2013) and Geneious version 11.0.4 (Biomatters Ltd, New Zealand).

The complete mitogenome sequence consist of 15,623 bp for *S. jaegeri* (Genbank: MH213061) and with the base composition A + T is 61.4%. Its genes’ arrangement and orientation are matched known Polydesmida mtDNA pattern (Dong et al. 2016). The typical ATG start codon is present in all PCGs except for *ND1* and *ND2* being with ATA and TTG, respectively. Two types of stop codons TAA and TAG are used for most genes, with the exception of incomplete stop codon T for *ATP6*, *ND1,* and *ND5.*

The two rRNA genes, 692 bp in *srRNA* and 1248 bp in *lrRNA*, are located between control region 1 (*CR1*) and *tRNA^Leu^(CUN)* and separated by *tRNA^Val^*. All the tRNA genes have typical cloverleaf secondary structures except for the *tRNA^Ser^(AGN)* and *tRNA^Ser^(UCN),* which lack the DHU arms. The 1017 bp long *CR1* is located between *tRNA^Ser^(UCN)* and *srRNA*, and the 181 bp long *CR2* are located between *tRNA^Thr^* and *tRNA^His^*, respectively. Of the genome sequenced here, no tandem repeat was found in these two regions.

To validate the phylogenetic analyses of *S. jaegeri* (Polydesmida), MrBayes (Ronquist et al. 2012) and RAxML (Stamatakis 2006) were used to reconstruct BI and ML tree, undering the best partitioned scheme and optimal model analyzed in Partitionfinder (Lanfear et al. 2012) (Model GTR + I + G). *Cermatobius longicornis* (Genbank: KC155628) and *Symphylella sp.* (Genbank: EF576853) were selected as out-groups. The phylograms obtained from BI and ML (data not shown) were all strongly indicated that Sphaerotheriida with an ancestral gene order of mitogenome for the Helminthomorpha was located in the basal branch (Gai et al. 2008; Dong et al. 2012). The topological structures between Spirobolida, Callipodida, Spirostreptida, Playtdesmida, and Julida were similar with that observed for other previously studies (Woo et al. 2007; Brewer et al. 2013). Extraordinarily, Julidae and Nemasomatidae were not clustered together, what is more, Xystodesmidae and Paradoxosomatidae were not a sister group in this study . These results provide novel molecular information that can potentially be used for phylogenetic of Diplopoda (Figure 1).

**Figure 1.**
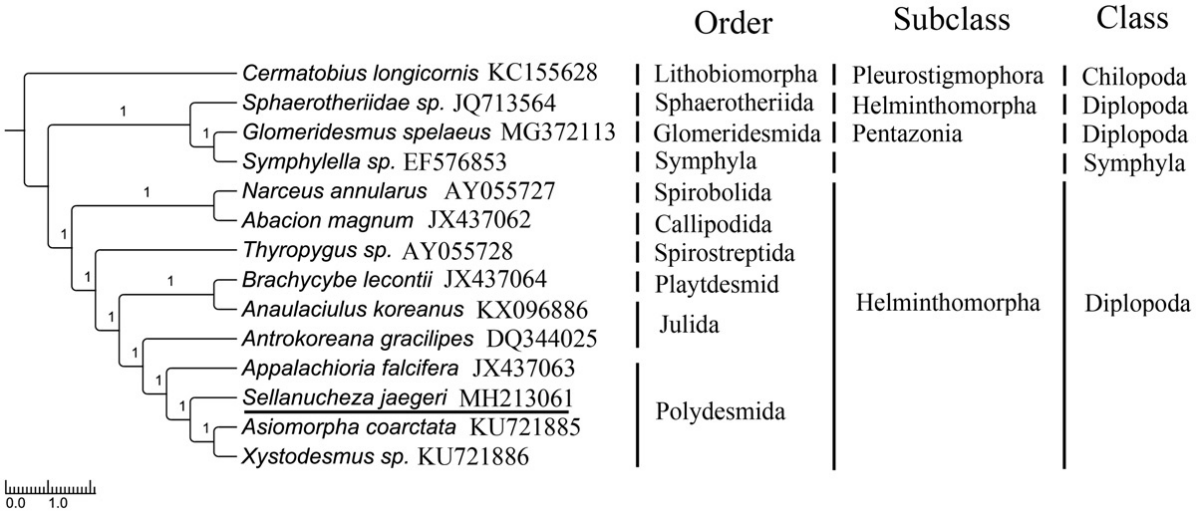
Topology of Bayesian tree for 14 species based on mitogenome PCGs sequences. GenBank accession numbers are indicated following species name. (Numbers on nodes are bootstrap values.)
